# First report of root-knot nematode, *Meloidogyne incognita*, infecting hops, *Humulus lupulus*, in São Paulo, Brazil

**DOI:** 10.21307/jofnem-2021-008

**Published:** 2021-03-01

**Authors:** R. F. Gonsaga, A. Souza Pollo, D. D. Nascimento, R. J. Ferreira, L. T. Braz, P. L. M. Soares

**Affiliations:** Department of Agricultural Production Sciences, School of Agricultural and Veterinarian Sciences, São Paulo State University (Unesp), Jaboticabal, São Paulo, Brazil

**Keywords:** First occurrence, Detection, Diagnosis

## Abstract

In 2019, during a nematologic survey in Jaboticabal, Brazil, root-knot nematode *Meloidogyne incognita* parasitizing hops (*Humulus lupulus*) was identified with based on morphological characters of adults, esterase phenotypes (*n* = 16), and molecular analysis. Modified Koch’s postulates was carried out and after 90 days, the average total population recovered had different stages of development, with a reproductive factor (RF) of 4.81. This is the first report of *H. lupulus* as a host of *M. incognita* in the state of São Paulo and in Brazil.

*Humulus lupulus* L. (Cannabaceae) is a creeping, herbaceous, perennial, and dioecious plant used mainly as a raw material in the beer brewing industry. Furthermore, this plant has medicinal properties and is used to treat insomnia, stress, and anxiety (Kyrou et al., 2017). *Humulus lupulus* has been cultivated in temperate regions for centuries, mainly in the Northern Hemisphere. However, after farmers efforts and management improvements, hop plants have been successfully cultivating in Brazil (Durello et al., 2019).

Hop seedlings of the Mantiqueira cultivar obtained from cuttings of vegetative parts of hop plants were planted in a field located in the region of Jaboticabal, Sao Paulo, Brazil (21°14′35.2″S 48°17′05.8″W) in October 2017. Seven months after transplanting, the plants presented symptoms such as yellow leaves, reduced development and root galls (Fig. 1A, B). Samples of hop roots and soil (*n* = 4) were collected and analyzed in the Nematology Laboratory (LabNema) of FCAV/UNESP according to the methodology proposed by Coolen and D’Herde (1972) and Jenkins (1964). The population number of nematodes encountered in 10 g of roots was 19,075 eggs and 2,675 *Meloidogyne* sp. individuals in different stages of development. In 100 cm^3^ of soil, the population of this nematode was 724 second-stage juveniles (J2s).

**Figure 1:**
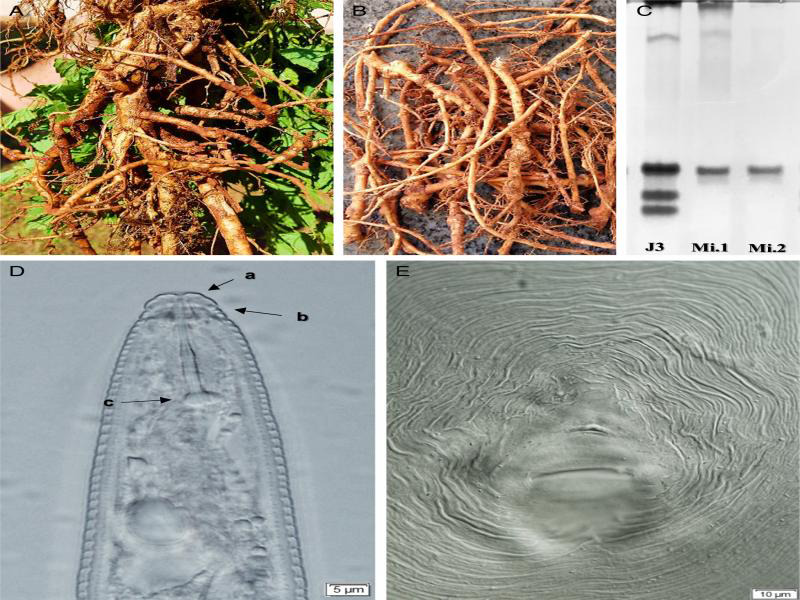
A. Hop roots, *Humulus lupulus* L. (Mantiqueira cultivar), with galls formed by *Meloidogyne incognita* (Kofoid and White, 1919) Chitwood, 1949; B. Roots with apparent nodules, characterizing the galls; C. Isoenzymatic esterase phenotype (I1 = *M. incognita*) of females recovered from hops roots Mi.1 and Mi.2; J3 = *M. javanica* (Treub, 1885) Chitwood, 1949, control. Trapezoidal labial region of male (a), a prominent labial disc in relation to the submedian lips with transverse streaks (b) and the stylet basal knobs height than wide (c); D. Perineal region of a female with high, trapezoidal dorsal arch and thick streaks, typical of *M. incognita*. São Paulo, Brazil.

The identification of the nematode characterized as *Meloidogyne incognita* (Kofoid and White, 1919) Chitwood, 1949, was based on morphological characters of adults, esterase phenotypes (*n* = 16), and molecular analysis. Morphological identification was performed by analyzing the male labial region, and presented a trapezoidal shape and prominent concave labial disk compared to the submedian lips and transverse striations on the head annule; the stylet basal knobs were higher than wide (*n*  = 12; Fig. 1D) (Eisenback and Hirschmann, 1981). The perineal region of females (Netscher and Taylor, 1974) presented a high, dorsal, and trapezoidal arch, with thick striations (*n* = 12; Fig. 1E) similar to the original description of the *M. incognita* species, enzymatic profile (esterase) obtained by electrophoresis resulted in phenotype I1 (Rm × 100 = 46.25), typical of *M. incognita* (Esbenshade and Triantaphyllou, 1985) (Fig. 1C). In addition, species identification was further confirmed by PCR with specific primers for *M. incognita* Finc/Rinc (Zijlstra et al., 2000) (Fig. 2).

**Figure 2:**
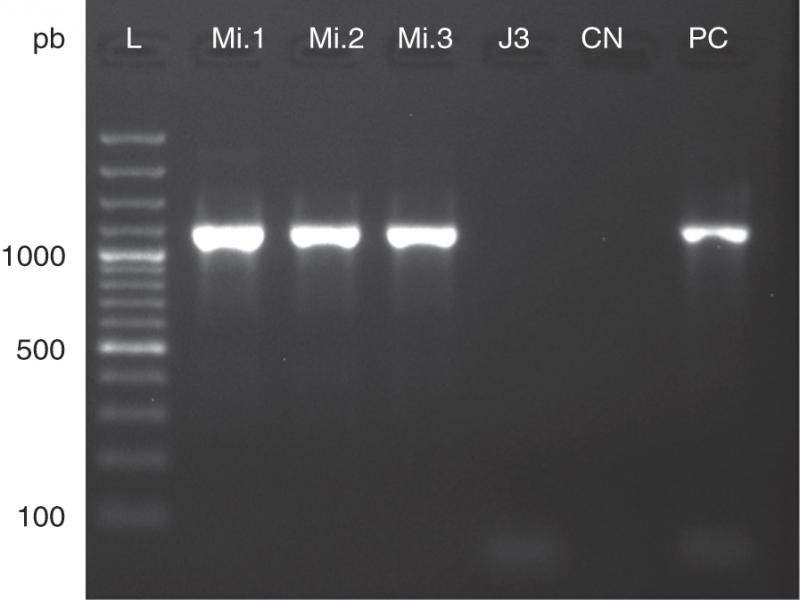
Molecular specific identification of *Meloidogyne incognita* (Kofoid and White, 1919) Chitwood, 1949, from roots of *Humulus lupulus* L. (Mi.1; Mi.2, Mi.3); L: 100 bp Plus DNA Ladder (Thermo Scientific); J3: negative control of *Meloidogyne javanica* (Treub, 1885) Chitwood, 1949, species; CN: negative control of PCR reaction; PC: positive control of *M. incognita*.

A modified version of Koch’s postulates was performed by inoculating 3,000 eggs and J2s of *M. incognita* in hop seedlings of the Mantiqueira cultivar (*n* = 4) transplanted in pots with autoclaved soil. The eggs and J2s were obtained from a pure population of *M. incognita* extracted directly from the roots (Coolen and D’Herde, 1972) of hop plants. The plants were kept from a greenhouse, and the average temperature observed in the period was 24.0°C, with a maximum and minimum of 30.7 and 19.1°C, respectively. After 90 days, the average total population recovered from the roots (Coolen and D’Herde, 1972) was 13,428 eggs and J2s, corresponding to a reproductive factor (RF) of 4.81 (Oostenbrink, 1966). Inoculated plants showed symptoms similar to those initially observed in the field, as yellowish leaves, reduced development, and a large number of galls on the roots, while uninoculated plants showed normal development, without root galls, confirming that the hops are host of *M. incognita*.

Nematodes in general have caused problems for the cultivation of hops in the world, be it *Meloidogyne* spp., *Heterodera humuli*, and *Ditylenchus destructor* (Darling et al., 2020; Foot and Wood, 1982; Lišková and Renčo, 2007; Renčo et al., 2010; Watson et al., 2020). There are reports of the occurrence of *M. incognita* in hops in Iran (Jamali et al., 2016), also presenting the typical symptom of root infestation. There have been reports of *Meloidogyne javanica* (Treub, 1885) Chitwood, 1949, causing damage to hops in Florida, USA and São Paulo, Brazil (Brito et al. 2018; Nascimento et al. 2020). Root-knot (*Meloidogyne* spp.) are problems in the main cultures of Brazil, causing serious damage and losses in several locations and cultures (Ferraz and Brown, 2016). *M. incognita* is a serious problem in crops such as maize, sugarcane, coffee, cotton, vegetables, and many others, being responsible for severe losses in Brazil (Ferraz and Brown, 2016).

Currently, there are few hop breeding programs in Brazil, one of them is located in Jaboticabal (Tiengo, 2019) where *M. incognita* was reported. So far there are no known hops cultivars with any resistance to gall nematodes. It is a great opportunity for breeding research, the search for new cultivars that are resistant to the most common nematodes in the tropical climate, as well as, discover from the existing cultivars what is the resistance level for the reported nematodes.

Based on all results, this is the first report of *Humulus lupulus* as a host of the root-knot nematode, *M. incognita*, in the state of São Paulo and in Brazil. Hops, in addition to being a perennial plant, are propagated vegetatively, which favors the spread of diseases, including those caused by nematodes. This report encourages the care to avoid the spread of this nematode to areas not yet infested and the search for new control strategies.
